# Novel Missense Variants in *PAX8* and *NKX2-1* Cause Congenital Hypothyroidism

**DOI:** 10.3390/ijms24010786

**Published:** 2023-01-02

**Authors:** Menglin Li, Zhuo Li, Miaomiao Chen, Zhiqing Hu, Miaojin Zhou, Lingqian Wu, Chunhua Zhang, Desheng Liang

**Affiliations:** Center for Medical Genetics & Hunan Key Laboratory of Medical Genetics, School of Life Sciences, Central South University, Changsha 410078, China

**Keywords:** congenital hypothyroidism, *PAX8*, *NKX2-1*, whole exome sequencing (WES), novel variants, functional study, dominant-negative effect

## Abstract

Primary congenital hypothyroidism (CH) is a common neonatal endocrine disorder characterized by elevated concentrations of thyroid stimulating hormone (TSH) and low concentrations of free thyroxine (FT4). PAX8 and NKX2-1 are important transcription factors involved in thyroid development. In this study, we detected three novel variants in *PAX8* (c.149A > C and c.329G > A) and *NKX2-1* (c.706A > G) by whole exome sequencing (WES) in three unrelated CH patients with variable phenotypes. The results of Western blot and immunofluorescence analysis showed that the three variants had no effect on protein expression and subcellular localization. However, the results of the electrophoretic mobility shift assay (EMSA) and dual-luciferase reporter assay suggested that the three variants in PAX8 and NKX2-1 both affected their DNA-binding ability and reduced their transactivation capacity. Moreover, a dominant-negative effect in K236E−NKX2-1 was identified by dual-luciferase reporter assay. To sum up, our findings extend our knowledge of the current mutation spectrum of *PAX8* and *NKX2-1* and provide important information for diagnosing, treating, and preventing CH in these families.

## 1. Introduction

Congenital hypothyroidism is the most common neonatal endocrine disorder worldwide, with a global prevalence of about 1 in 2000–4000 newborns [1]. Congenital hypothyroidism in early infancy leads to irreversible intellectual disability and physical development retardation if left untreated [2,3].

Primary congenital hypothyroidism (CH) is diagnosed based on elevated TSH concentrations and low concentrations of free thyroxine (FT4), and hyperthyrotropinemia (HT) is defined as having high TSH and normal FT4. In recent years, the incidence of CH caused by dyshormonogenesis has increased to 40–50% compared to the previous reported level of 15–25%, while the remaining 50–60% is due to thyroid dysgenesis (TD) [4,5,6,7]. With the high coverage of neonatal screening, CH patients can be diagnosed at a very early stage and receive timely therapy with thyroid hormone replacement with levothyroxine (LT4) [8,9]. However, it is insufficient to rely on clinical biochemical examination and thyroid ultrasound to give the prognosis of CH and formulate long-term treatment plans.

The thyroid gland is the body’s first endocrine gland to develop, which arises from the pharyngeal pouches [10]. The known genes associated with thyroid development are *PAX8*, *NKX2-1*, *NKX2-5*, *FOXE1*, *GLIS3*, and *TSHR*. Among them, the transcripts of *NKX2-1* are expressed in the median thyroid anlage, lung bud, and forebrain areas at days 32 and 33 in human embryonic development. The expression of PAX8 can be strongly detected in the median anlage and the fourth pharyngeal arch ectoderm by day 32, and it maintains a strong expression during thyroid development [10]. With the development of the thyroid, the foregut endoderm cells differentiate a specific follicular linage—thyroid follicular epithelial cells (TFCs). Through cell polarization, the follicles are formed by TFCs, and the thyroid follicles are the basic unit of the thyroid [3,11]. Thyroid hormone (TH) is synthesized by thyroid follicle epithelial cells. After a series of processes including iodine trap, iodization, and condensation, it is stored in the follicles and released into the blood under the control of thyroid-stimulating hormone (TSH) [9]. Several genes have been implicated in hormone synthesis, including *DUOX2*, *DUOXA2*, *TG, TPO, SLC5A5, SLC26A4, SLC25A7*, and *IYD* [12,13,14].

The advance of next-generation sequencing (NGS) is conducive to identifying the genetic factors of CH and deepening our insight into the molecular cause of CH. Whole exome sequencing (WES) can help us to identify mutations occurring in exons and splicing sites to a relatively high degree of accuracy. Understanding the association between genotype and phenotype will help us in the accurate diagnosis of CH, creation of individualized treatment, and providing reproductive guidance.

In this study, the genetic characteristics of three unrelated CH patients with variable phenotypes were analyzed based on the results of the WES. Several mutations associated with CH were detected, among which the pathogenicity of novel mutations in *PAX8* and *NKX2-1* were determined by corresponding functional experiments.

## 2. Results

### 2.1. Clinical Presentation

The clinical information is recorded briefly in Table 1.

P1 was a full-term female neonate with a birth weight of 3550 g, showing a thyroid gland with small diameter. The TSH value at the time of newborn screening was as high as 269 mU/L (TSH screening reference values <10 mU/L). CH was confirmed at the age of 31 days, with a TSH of 100 mU/L (TSH screening reference values in confirmatory test, 0.35–5.5 mU/L) and fT4 values of 5.02 pmol/L (fT4 reference values for neonates, 11.21–22.65 pmol/L). Urinary ultrasound did not reveal any anomalies at 4 years of age.

P2 was a full-term female infant with a birth weight of 2600 g. She was diagnosed as having persistent hyperthyrotropinemia (PHT) and had a normal thyroid size according to ultrasound. The TSH value at the time of newborn screening was 131 mU/L, and this decreased to 10.7 mU/L by the time of a confirmatory test (39 days), with a normal level of fT4 (16.9 pmol/L). We were unable to track the medication of P1 and P2 due to their therapy being conducted at other local hospitals.

P3 was a full-term female with a birth weight of 3400 g who exhibited a small-sized thyroid gland (Figure S1). The TSH value at the time of newborn screening was 14.4 mU/L, and she was diagnosed as CH in a confirmatory test (41 days), with the TSH value of 36.34 mU/L and the fT4 value of 11.96 pmol/L, (very close to the lower limit of the reference range). It is worth noting that the girl suffered recurrent pneumonia after 6 months of age, and markedly delayed motor development and language development were observed after 9 months of age. Multiple development-related indicators showed markedly delayed development of motor and language skills at 5 years old (Figure S2).

### 2.2. Identification of Variants

**Figure 1:**
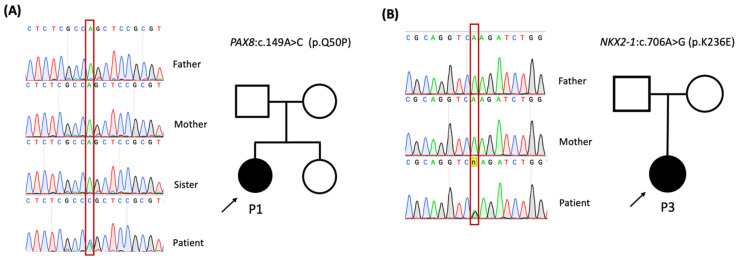
Sanger validation of the variants in the probands and their parents. Arrows indicate the probands. (**A**) The variant of *PAX8*:c.149C > A in the P1. (**B**) The variant of *NKX2-1*:c.706A > G in the P3.

The WES detected eight heterozygous missense variants in six CH-related genes among our patients (Table 2 and Table 3). Variants in *PAX8* and *NKX2-1* were suspected to cause CH. P1 carried the novel heterozygous *PAX8* variant (c.149A > C p.Q50P), which is a de novo variant that was not inherited from her parents (Figure 1A). This variant is classified as “likely pathogenic” following the American College of Medical Genetics and Genomics (ACMG) criteria. P2 presented with novel heterozygous *PAX8* variant (c.329G > A p.R110Q), which is classified as “variant of uncertain significance”. Unfortunately, we could not obtain blood samples from the family members of P2 for family co-segregation analysis. The genetic study showed P3 carried the novel heterozygous *NKX2-1* missense variant (c.706A > G p.K236E), which is also a de novo variant and classified as a variant of uncertain significance (Figure 1B).

### 2.3. In Silico Studies

**Figure 2:**
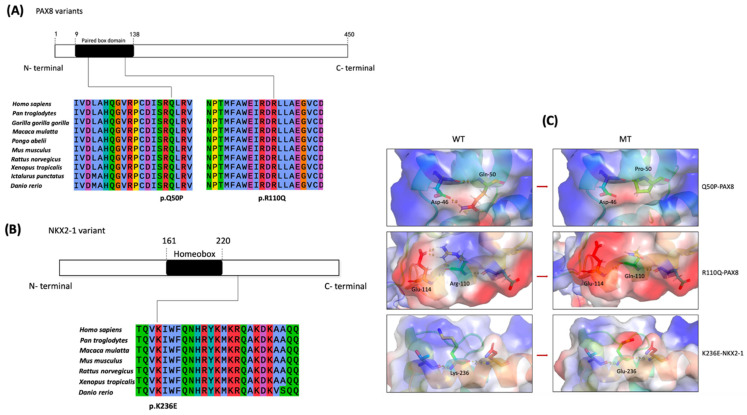
Location diagram and conservation analysis of PAX8 variants and NKX2-1 variants. (**A**) The Q50 and R110 residues of PAX8 located at the paired box domain were highly conserved across various species. (**B**) The K236 residues of NKX2-1 was highly conserved across various species. (**C**) 3D structural model of wild-type and mutant proteins. Amino acids are displayed using a color rod structure. Hydrogen bonds between amino acids are marked by yellow dashed lines. In the upper level are WT−PAX8 and Q50P−PAX8; in the medium level are WT−PAX8 and R110Q−PAX8; in the bottom level are WT-NKX2-1 and K236E−NKX2-1. The surface charge distribution is shown in the outer layer; blue represents a positive charge, red represents a negative charge, and white represents a neutral charge.

The variants of PAX8 (Q50P and R110Q) occurred in the paired box domain that is highly conserved across species; this is considered as an important functional domain for DNA binding (Figure 2A). K236E−NKX2-1 was located in the C-downstream of the homeobox domain, which is highly conserved among species (Figure 2B).

Three-dimensional structural models were built in order to visualize the structural changes in nearby protein regions after amino acid changes (Figure 2C). The variants Q50P−PAX8 and R110Q−PAX8 were located at the second α-helix region and the fifth α-helix region, respectively, of the paired-box domain. It was observed that hydrogen bonds between glutamine at position 50 and aspartic acid at position 46 were lost in the Q50P mutant model. Two hydrogen bonds between arginine at position 110 and glutamate at 114 were lost in the R110Q mutant model. No hydrogen bond change was observed in K236E−NKX2-1. Furthermore, the change in the protein surface charge was predicted in the three mutant protein models.

### 2.4. The Three Variants Showed Normal Expression Level and Localization

**Figure 3:**
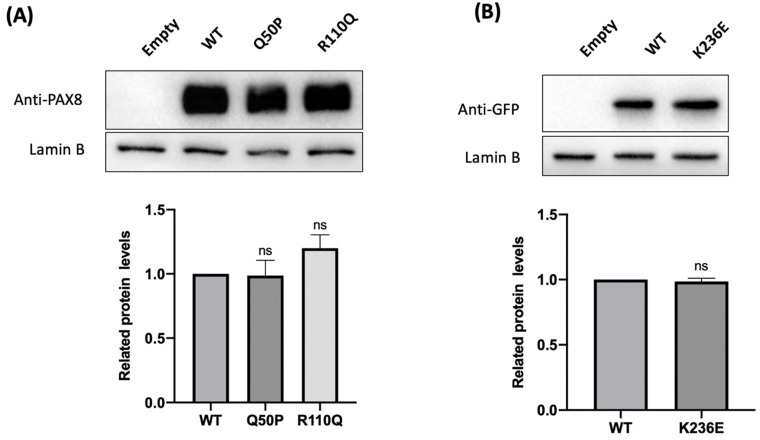

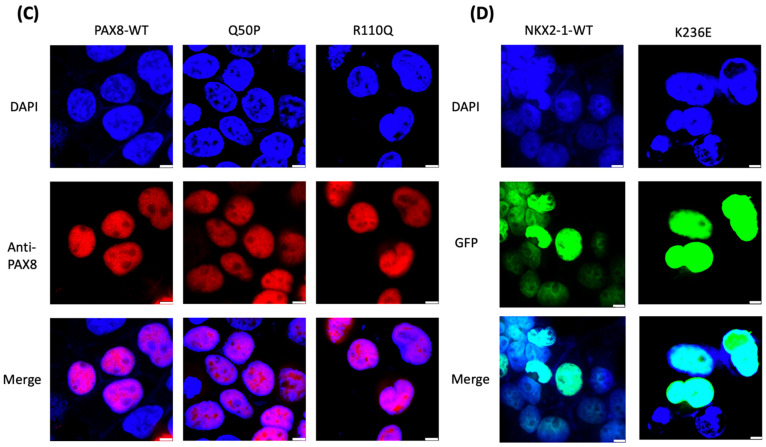
Western blot analysis and immunofluorescence localization of the wild-type and mutant PAX8 and NKX2-1. (**A**) No statistically significant difference in normalized amounts was seen between the wild-type and mutant proteins. The expression was quantified as the gray scale ratio of PAX8/Lamin B. (Mann–Whitney test, ns: *p* > 0.05) (**B**) NKX2-1−GFP fusion proteins were detected using anti-GFP antibody. No statistically significant difference in normalized amounts was seen between wild-type and mutant proteins. The expression was quantified as the gray scale ratio of GFP/Lamin B. The results of three independent experiments were compared for analysis (Mann–Whitney test, ns: *p* > 0.05). (**C**) Subcellular localization of PAX8−WT, Q50P, and R110Q were examined using confocal microscopy; nuclei were identified by DAPI staining. Analysis of the subcellular localization showed that 2 variants were predominantly localized to the nucleus, similar to WT. (**D**) Analysis of the subcellular localization of NKX2-1−GFP showed that K236E was predominantly localized to the nucleus, similar to WT. The scale bars indicate 5 μm.

Western blot was applied to investigate whether the mutants affected the expression level of the protein. There was no significant decrease in the expression levels of Q50P-PAX8, R110Q−PAX8, and K236E−NKX2-1 compared to the wild type. Cells with empty vector pCMV3 were used as the negative control group. Moreover, we found that PAX8 and NKX2-1 were not expressed endogenously in 293 T cells (Figure 3A,B).

Given that the mutants may cause the abnormal subcellular localization of the protein, further immunofluorescence analysis was performed. The results showed that the variants Q50P−PAX8, R110Q−PAX8, and K236E−NKX2-1 were mainly located in the nucleus compared with the wild type (Figure 3C,D). These results suggested that the variants did not result in the abnormal subcellular localization of PAX8 and NKX2-1.

### 2.5. Variants Affected the Ability of DNA Binding and Transactivation

**Figure 4:**
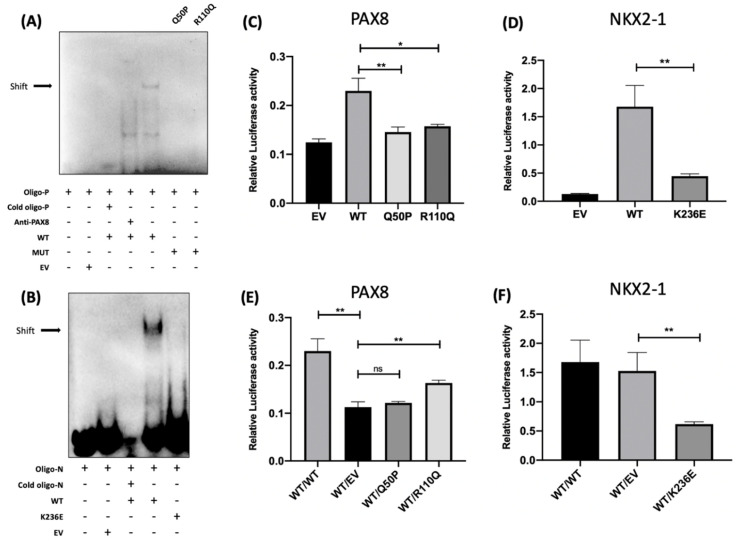
Results of EMSA and dual-luciferase reporter assay (EV: empty vector for negative control). (**A**) EMSA of PAX8: Lane 1, EMSA with oligo only; Lane 2, EMSA with empty vector transfected control extract; Lane 3, EMSA with competitive oligo and PAX8-WT; Lane 4, super shift EMSA with PAX8−WT and anti-PAX8 antibody; Lane 5, EMSA with oligo and PAX8−WT; Lane 6, EMSA with oligo and PAX8−Q50P; Lane 7, EMSA with oligo and PAX8−R110Q. Lane 5 showed a specific binding shift. (**B**) EMSA of NKX2-1: Lane 1, EMSA with oligo only; Lane 2, EMSA with empty vector transfected control extract; Lane 3, EMSA with competitive oligo and NKX2-1−WT; Lane 4, EMSA with oligo and NKX2-1−WT; Lane 5, EMSA with oligo and NKX2-1−K236 E (*t* test, ns: *p* > 0.05; *: *p* < 0.05; **: *p* < 0.01). (**C**) *TG* transactivation by PAX8−WT and PAX8 mutants was assessed; the transactivation ability of Q50P and R110Q to *TG* promoter was shown to decrease. (**D**) Co-transfection of PAX8−WT and variants (200 ng in total). (**E**) Transactivation activity of NKX2-1 on *TG* promoter; a significant decrease was observed for K236E−NKX2-1. (**F**) Co-transfected NKX2-1−WT and K236 E (200 ng in total). K236 E exerted a dominant-negative effect. The data shown represent the mean ± SEM of at least three independent experiments (each performed in triplicate) (*t* test, *: *p* < 0.05; **: *p* < 0.01).

Subsequently, EMSA experiments were conducted to evaluate the DNA binding ability of the mutant protein. The results showed that the WT−PAX8 and WT−NKX2-1 can both specifically bind to DNA, and this binding was competed by a 20-fold excess cold competitor (Figure 4A,B). In contrast, the DNA binding ability of Q50P−PAX8 and R110Q−PAX8 was abrogated (Figure 4A). K236E−NKX2-1 lost its binding ability as well (Figure 4B).

The transactivation ability of the mutant protein to the *TG* promoter was also investigated via dual-luciferase reporter assay. As compared to PAX8−WT, the transactivation ability of Q50P and R110Q to *TG* promoter decreased by 46.4% and 38.9%, respectively (Figure 4C). Compared with WT−NKX2-1, a more significant decrease (73.5%) was observed for K236E−NKX2-1 (Figure 4D).

### 2.6. Dominant-Negative Effect Was Found in K236E-NKX2-1

In order to explore whether the mutant protein has a dominant negative effect, we co-transfected WT−PAX8/pCMV3, WT−PAX8/Q50P, WT−PAX8/R110Q, WT−NKX2-1/pCMV3, and WT−NKX2-1/K236E. The total amount of transfected plasmid was equalized for all strategies. It was found that Q50P−PAX8 and R110Q−PAX8 had negligible effects on the transactivation ability of WT, indicating that the two missense variants of PAX8 exerted no dominant-negative effect (Figure 4E). However, the transactivation ability of the *TG* promoter under the co-transfection of K236E−NKX2-1 with WT was significantly lower than that of WT−NKX2-1 transfected alone, suggesting that the mutant protein affected the transactivation ability of the WT protein and exerted a dominant-negative effect (Figure 4F).

## 3. Discussion

The protein encoded by *PAX8* (OMIM 218700, 2q12−q14) is a transcription factor belonging to the paired box (PAX) family. The *PAX8* gene was first revealed to participate in thyroid gland development in 1990. PAX8 protein plays an important role in thyroid formation and maintaining the normal morphology of thyroid cells [15,16]. This protein has a highly conserved paired-box domain, which can combine the promoter sequence of the *TG*, *TPO*, and *NIS* genes and regulate the transcription and expression of the target genes. *PAX8* is expressed in other organs during embryogenesis as well [17], and renal hypoplasia has been reported in a CH patient with *PAX8* variants [18], but no extra-thyroid phenotype was found in our patients.

The phenotypic heterogeneity caused by PAX8 variation is remarkable; the two variants p.Q50P and p.R110Q we identified were both located in a highly conserved paired-box domain, but the patients P1 and P2 showed variable clinical and biochemical phenotypes. Compared with the relatively mild phenotype of P2, P1 was diagnosed with severe CH with thyroid dysplasia. Our experiment data clearly showed that the variant p.Q50P of P1 is deleterious. The protein structural model shows that when the 50 Gln mutates into Pro, the hydrogen bond originally linked to the amino acid residue of 46 Asp disappears, which may have a remarkable impact on the α-helix structure and cause functional changes. However, the mutant p.R110Q only lost part of its hydrogen bonds. We speculate that such a change has less effect on the structure of the α-helix. The experimental results also show that p.R110Q experienced less decrease in its transactivation ability than p.Q50P. These results may explain the milder phenotype of P2. The results of the EMSA showed that p.Q50P and p.R110Q did not have a binding band; correspondingly, the transactivation ability of the mutant was significantly decreased compared with the wild type, which also proved the pathogenicity of the two variants. Due to the weaker capability of the EMSA for quantitative detection, the reduction in its binding capacity may lead to there being no binding band, while the dual-luciferase reporter assay can quantitatively show the reduction of transactivation.

It is worth noting that P2 also presented with two heterozygous variants in *SLC26A4* (c.1983C > A and c.853G > A), but we cannot confirm whether the two variants were in cis or in trans, since the parents of P2 refused to provide their DNA samples. The homozygous or compound heterozygosity of pathogenic variants in *SLC26A4* cause Pendred syndrome, which is described as including sensorineural hearing loss, developmental malformations of the inner ear, and thyroid dyshormonogenesis [7]. Follow-up revealed that P2 and her parents had no related phenotypic involvement, but we informed the family about the risk of Pendred syndrome and recommended referral to otolaryngology. Moreover, the heterozygous variant *SECISBP2*:c.1698T > A was detected in P2. SECISBP2 is an essential factor for selenoprotein synthesis, affecting thyroid hormone metabolism by regulating the synthesis of iodothyronine deiodinase [19]. *SECISBP2* variants may cause abnormal thyroid hormone metabolism; however, all reported affected cases include homozygous or complex heterozygotes [19,20,21,22,23,24,25]. In P2, HT may have an oligogenic origin, and the variants in *PAX8*, *SLC26A4*, and *SECISBP2* may be involved in the pathogenesis of HT. After determining the pathogenicity of *PAX8* variant, follow-up will be maintained to observe the impact of other mutations on the disease.

Thyroid transcription factor-1 (TTF-1/NKX2-1) protein is encoded by *NKX2-1* (OMIM 600635, 14q13.3). NKX2-1, which has a key homeobox domain, participates in the development of thyroid, lung, forebrain regions, basal ganglia, and hypothalamus [26]. In thyroid tissues, NKX2-1 cooperates with PAX8, has a transactivation effect on *TG* promoter, and regulates the expression of surfactant protein-B (SP-B) and surfactant protein-C (SP-C) in lung tissues [27]. Pathogenic variants in the *NKX2-1* gene could cause brain–lung–thyroid syndrome (BLTS) [28,29,30,31,32].

According to NBS and confirmatory tests, P3 was diagnosed as CH and L-T4 therapy was initiated timely. However, the patient showed global developmental delay, dysarthria, ataxia, and recurrent pneumonia during subsequent follow-up. Diagnosis of BLTS was not made until the variant *NKX2-1*:c.706A > G was identified by WES. *FOXE1*: c.571C > T and *SLC26A7*: c.1074C > G were also detected in P3; however, these two genes cause CH in autosomal recessive inheritance pattern and the variants were classified as VUS. The variant *NKX2-1*:c.706A > G was novel and there was a lack of the pathogenicity evidence needed for genetic diagnosis; thus, an in vitro functional experiment was conducted. Then, the results of EMSA and dual-luciferase reporter assay demonstrated that the primary reason for the impaired function of the mutant protein was the weaker DNA-binding capacity and reduced transactivation ability of the *TG* promoter. Based on our findings, the patient was diagnosed as having brain–lung–thyroid syndrome, and the personalized therapeutic regimen, including L-T4 and rehabilitation training, was scheduled to optimize the outcome of the patient.

Moreover, the dual luciferase reporter assay showed that K236E−NKX2-1 significantly reduced the transactivation ability of wild-type NKX2-1 to *TG* promoter. Up to now, more than 100 variants of NKX2-1 have been reported, and the pathogenesis of most variants in NKX2-1 was attributed to haploinsufficiency. However, a dominant-negative effect was observed in K236E. Only six NKX2-1 variants have been reported to have a dominant-negative effect on the *TG* promoter, including three missense variants (L176V, R178P, and I207F) and three frameshift variants (R165fs, L263fs, and P275fs) [28,30,33,34,35,36]. In our 3D protein structure model, although the variant did not affect the stability of the helix structure, E236 caused an abnormal surface charge distribution. Changes in molecular mechanics may cause multiple effects, which may result in abnormal binding with other cofactors in the preinitiation complex, thus affecting the trans activation ability of the wild type of NKX2-1, but the specific mechanism involved still needs further investigation [37]. In addition, Li et al. reported the dominant negative effect of NKX2-1−Lys182 acetylation site mutation on the transcription of *SP-B* gene, which supported the important effect of lysine acetylation in the conservative sequence on NKX2-1 transcription activity, which may partly explain why our K236E has a dominant negative effect [38]. Our findings confirmed the functional impairment of K236E−NKX2-1, providing a reference for other researchers to expand their studies on the more complex pathogenesis of NKX2-1.

Several CH-related genes have been identified so far; the detection rate of pathogenic mutations in CH patients has increased with the inclusion of more genes in NGS-panel [39,40,41]. WES can help to identify the pathogenic mutations in CH-related genes to greater extent and even provides the possibility to search for novel causative genes for CH [42,43,44]. More significantly, the identification of causative genes can clarify the molecular mechanism of CH, and the detection of genes associated with complex syndrome is conducive to intervention in the extra-thyroid phenotype of patients in a timely manner.

In summary, we identified two *PAX8* variants (c.149A > C and c.329G > A) and a *NKX2-1* variant (c.706A > G) from three CH patients with different phenotypes. All of these were heterozygous missense variants which have not been previously described. The mutation spectra of *PAX8* and *NKX2-1* were enriched. Combined with the experimental data, the classification of three variants could be upgraded to “likely pathogenic” or “pathogenic”. Our findings contribute to the genetic diagnosis of patients with CH, help to improve their clinical follow-up, and provide a basis for long-term individualized treatment.

## 4. Materials and Methods

### 4.1. Patients and Clinical Information

Patients’ blood samples were collected after obtaining written informed consent from their guardians. Their medical records were extracted from the newborn screening (NBS) information system (data collection ended on 30 September 2020). Gestational weeks, birth weight, length, thyroid ultrasound, and TSH level at the newborn screening of P1-P3 are recorded in Table 1. The TSH level and FT4 level at reevaluation are covered as well. Additionally, an evaluation of behavior development was taken for P3 (Figure S2).

### 4.2. Whole Exome Sequencing and Mutation Detection

In our previous study, we sequenced the whole exons of 233 patients with CH (data not yet published). Genomic DNA was extracted from dried blood spots (DBSs) with the Blood gDNA Kit (RC1002, Concert Biotech, Xiamen, China). Exome enrichment and library preparation were performed using the Human Whole Exome Detection Kit (KR9011, Berry Genomics, Beijing, China). A capture rate of 95% and a duplication rate of 30% were achieved. Next, the candidate variants associated with CH were screened. The screening process was based on sequencing depth, population frequency, software prediction and phenotype correlation. Additionally, the dried blood spots of some family members were collected as DNA sources for Sanger sequencing (Tsingke Biotechnology Co., Ltd., Beijing, China).

The variants with a minor allele frequency (MAF) <0.1% in the Exome Aggregation Consortium (ExAC), the 1000 Genomes project (1000 Genomes), and the Genome Aggregation Database (gnomAD) were filtered in the first step. Then, the candidate variants associated with hypothyroidism were screened. Multiple bioinformatics methods were used to predict the pathogenicity of candidate variants, including Polyphen-2 (http://genetics.bwh.harvard.edu/pph2/, accessed on 20 August 2021), SIFT (http://blocks.fhcrc.org/sift/SIFT.html, accessed on20 August 2021), MutationTaster (http://www.mutationtaster.org/, accessed on 20 August 2021), PROVEAN (http://provean.jcvi.org/index.php, accessed on 20 August 2021), ClinPred (https://sites.google.com/site/clinpred/, accessed on 20 August 2021), REVEL (https://sites.google.com/site/revelgenomics/, accessed on 20 August 2021), GERP (http://mendel.stanford.edu/SidowLab/downloads/gerp/, accessed on 20 August 2021), and MutationAssessor (http://mutationassessor.org/r3/, accessed on 20 August 2021) (Table S1).

### 4.3. In Silico Studies

The sequences of *PAX8* (MN_003466.3) and *NKX2-1* (NM_001079668.2) were obtained from the NCBI (National Center for Biotechnology Information, https://www.ncbi.nlm.nih.gov/, accessed on 20 August 2021) database and UCSC (University of California Santa Cruz, http://www.http://genome.ucsc.edu/index.html, accessed on 20 August 2021) database. The functional domain of PAX8 was predicted according to the literature [14]. The amino acid sequences of different species were obtained from NCBI and compared with the SnapGene software to analyze their conservation. The Swiss model (https://swissmodel.expasy.org, accessed on 20 August 2021) was used to build a homology model of the wild type of PAX8 and NKX2-1. PyMOL (The PyMOL Molecular Graphics System, Schrödinger, LLC., New York, NY, USA) was used for mutation and molecular visualization.

### 4.4. Plasmids and Cell Culture

In vitro functional experiments were designed to verify the pathogenicity of *PAX8* and *NKX2-1* variants. Wild-type expression vectors (PAX8−WT−pCMV3, NKX2-1−WT−pCMV3) were purchased from Sino Biological (Sino Biological Inc., Beijing, China). Mutant vectors (PAX8−Q50P−pCMV3, PAX8−R110Q−pCMV3, and NKX2-1−K236E−pCMV3) were constructed using the Mut Express II Fast Mutagenesis Kit V2 (Vazyme Biotechnology Co., Ltd., Nanjing, China) through a PCR-based site-specific mutagenesis strategy. The GFP vector (NKX2-1−GFP−pcDNA3.1 and NKX2-1−K236E−GFP-pcDNA3.1) was constructed using restriction endonuclease EcoRI (New England Biolabs, Beijing, China) and the ClonExpress II One Step Cloning Kit (Vazyme Biotechnology Co., Ltd., Nanjing, China). The *TG* Promoter sequence (−322/+89) was cloned into the luciferase reporter vector (pGL3−basic, Promega, Madison, WI, USA) [15]. All the specific primers were ordered from Tsingke (Tsingke Biotechnology Co., Ltd., Beijing, China) (Table S2). The plasmids were extracted using the Endo-Free Plasmid DNA Maxi Kit (Omega Bio-Tek Inc., Guangzhou, China), and the sequences of all vectors were verified by Sanger sequencing prior to cell transfection. Then, the plasmids were extracted using the Endo-Free Plasmid DNA Maxi Kit (Omega Bio-Tek Inc., Guangzhou, China).

HEK293T human cell lines were cultured for Western blots, immunofluorescence, electrophoretic mobility shift assay (EMSA), and dual-luciferase reporter assay. Briefly, cells were inoculated in DMEM supplemented with 10% fetal bovine serum (GIBCO, New York, NY, USA) and cultured in 37 °C and 5% CO_2_, and cells were transfected with wild-type or mutant plasmids using LipofectamineTM3000 Transfection Reagent (Thermo Fisher Scientific, Shanghai, China).

### 4.5. Western Blot and Immunofluorescence

For Western blot analysis, 293 T cells were seeded in 6-well plates at a level of 5 × 10^5^ per well and transfected with 2.5 μg plasmid per well. After 48 h, each sample was prepared for the 2X SDS solution with PMSF, and then we added loading buffer after boiling for 10 min. The protein was separated on a 10% sodium dodecyl sulfate-polyacrylamide gel and then transferred to polyvinylidene fluoride (PVDF) membranes for Western blot. Here, anti-PAX8 antibody (1:4000, ab191870, Abcam, Cambridge, UK) and anti-GFP antibody (1:10000, GTX113617, GeneTex Inc., Irvine, CA, USA) were used as the primary antibodies for PAX8 and NKX2-1, respectively. After stripping the primary antibodies, the internal reference was diluted with anti-Lamin B antibody (1:10000, Proteintech, Beijing, China). Images were captured using a Chemiluminescence Imaging System (Bio-red Molecular Imager ChemiDocTM XRS+, CA, USA).

For immunofluorescence analysis, 293 T cells were grown on 24-well dishes and transfected with 500 ng plasmid per well when cells were cultured to 50–60% confluence. Upon 48 h, cells washed twice with PBS were fixed in 4% paraformaldehyde for 30 min, permeabilized in PBS containing 0.5% Triton X-100 for 20 min, and finally blocked in 5% BSA for 30 min. Then, the cells were incubated with primary antibody for 12 h at 4 °C, secondary Dylight 488 Rabbit Anti-Goat antibody for 2 h, and DAPI for 10 min. Immunofluorescence imaging was observed by TCS SP5 laser confocal microscopy (Leica, Wetzlar, Germany).

### 4.6. Electrophoretic Mobility Shift Assay

For EMSA, 293 T cells were transfected with untagged expression vectors. After 48 h, the nuclear protein was isolated with the Nuclear and Cytoplasmic by Protein Extraction Kit (Beyotime Biotechnology Co., Ltd., Shanghai, China). The design and synthesis of biotin-modified probes were based on JASPAR (https://www.jaspar.genereg.net, accessed on 14 December 2021) predictions and the previous literature [29]. The sequence of the *TG*-probe for PAX8 was 5-TCCTCATGCTCCACTGGCCA-3, and that for NKX2-1 was 5-TCAGGACACACAAGAGGCCCGGCGC-3. Each reaction contained 4 μg protein and 500 fmol probe. For competition experiments, a large excess (20 times) of unlabeled competitor oligonucleotides was included in the binding reactions. The anti-PAX8 antibody was used to distinguish the specific binding shift. A protein-free sample and a protein-containing sample from empty vector-transfected cells served as negative control groups.

Chemiluminescence imaging was performed with the Chemiluminescent EMSA Kit (Beyotime Biotechnology Co., Ltd., Shanghai, China) and Chemiluminescence Imaging Systems (Bio-red Molecular Imager ChemiDocTM XRS+).

### 4.7. Dual-Luciferase Reporter Assay

For the dual-luciferase reporter assay, the cells were inoculated one day before transfection on 24-well plates. TG-pGL3 reporter vector (300 ng/well), renilla fluorescein reporter vector pRL-TK (Promega, Madison, WI, USA) (5 ng/well), and expression vector (200 ng/well) were transiently co-transfected. After transfection for 48 h, cells were washed with phosphate buffered saline and lysed with 100 μL 5× PLB buffer for 30 min in the dark. The chemiluminescence signal was read on the multifunctional microplate reader (spectramax ID3) using the Dual-Luciferase Reporter (DLR) Assay System (Promega, Madison, WI, USA), and the reading of pRL-TK carrier renilla fluorescein was used as the internal reference.

### 4.8. Statistical Analysis

Statistical analyses were performed using SPSS (IBM Corp) and Prism 9 (Graph-Pad, La Jolla, CA, USA).

## Figures and Tables

**Table 1:** Clinical information of the patients.

Patient	YOB, Sex	Newborn Screening	Age at Diagnosis, d	Confirmatory Test		Thyroid Ultrasound	Diagnosis
		TSH, mU/L (<10)		TSH, mU/L (0.35−5.5)	fT4, pmol/L (11.21−22.65)		
P1	2016, female	269.00	31	>100	5.02	Small thyroid	CH
P2	2018, female	131.00	39	10.70	16.90	Normal	PHT
P3	2016, female	14.40	41	36.34	11.96	Small thyroid	CH

YOB, year of birth. The final diagnosis was based on TSH and FT4 levels at the time of the confirmatory test. The instruments used for confirmatory test cannot measure values higher than 100 mU/L, The reference range of TSH in screening, TSH and fT4 in confirmatory test are noted in parentheses.

**Table 2:** Potential pathogenic variants in our patients.

Patient	Gene	Localization	Nucleotide Substitutions	Amino Acid Change	Population Frequency (ExAC or 1000 G)	ACMG Classification
P1	*PAX8*	Exon 3, Paired box domain	c.149A > C	p.Q50P	0	LP (PS2_Moderate, PM1, PM2_Supporting, PP3)
P2	*PAX8*	Exon 4, Paired box domain	c.329G > A	p.R110Q	0	VUS (PM1, PM2_Supporting, PP3)
P3	*NKX2-1*	Exon 4	c.706A > G	p.K236E	0	VUS (PS2_ Moderate, PM2_Supporting, PP3)

*PAX8*: MN_003466.3, *NKX2-1*: NM_001079668.2. ACMG, American College of Medical Genetics. LP, likely pathogenic. VUS, variant of uncertain significance.

**Table 3:** Other variants in CH related genes in our patients.

Patient	Gene	Localization	Nucleotide Substitutions	Amino Acid Change
P2	*SLC26A4*	Exon 17,	c.1983C > A	p.D661Q
P2	*SLC26A4*	Exon 7,	c.853G > A	p.V285I
P2	*SECISBP2*	Exon 42	c.1698T > A	p.R2455H
P3	*FOXE1*	Exon 1	c.571C > T	p.P191S
P3	*SLC26A7*	Exon 10	c.1074C > G	p.F358L

## Data Availability

The data that support the findings of this study are available from the corresponding author upon reasonable request.

## Supplementary Materials

The following supporting information can be downloaded at https://www.mdpi.com/article/10.3390/ijms24010786/s1.
