# Supplementary material for: Novel Missense Variants in PAX8 and NKX2-1 Cause Congenital Hypothyroidism
Source: Int J Mol Sci. 2023 Jan 2;24(1):786. doi: 10.3390/ijms24010786 (PMC9821711; doi:10.3390/ijms24010786)
Supplement: Supplementary file 1 [file ijms-24-00786-s001.zip › ijms-1984221-supplementary.pdf]

Table S1. Function and conservative prediction of variants.

Variants	Polyphen-2_HVAR	SIFT	MutationTaster	PROVEAN	ClinPred	REVEL	GERP++	MutationAssessor
PAX8: c.149A>C	Probably_ damaging	Damaging	Disease_causing	Damaging	pathogenic	Damaging	Conserved	High
PAX8: c.329G>A	Probably_ damaging	Damaging	Disease_causing	Damaging	pathogenic	Damaging	Conserved	Low
NKX2-1: c.706A>G	Probably_ damaging	Damaging	Disease_causing	Damaging	pathogenic	Damaging	Conserved	Medium

Table S2. Primers used for the expression vectors and thyroglobulin (TG)-promoter cloning.

Vector	Forward primer	Reverse primer	Variant/Insert
TG-pGL3	GATCTGGATCTAAGTAAGCTTCAGGCAG AGTGGATCTGAGC	CAGTACCGGAATGCCAAGCTTTTCCTG GGAGGAAGGAGGAG	TG promoter sequence (-411/+89)
PAX8-c149- pCMV3	ACATCTCTCGCCCGCTCCGCGTCAGCCA TGGC	GAGCGGGCGAGAGATGTCGCAGGGCC TTACAC	c.149A>C (p.Q50P)
PAX8-c329- pCMV3	ATCCGAGACCAGCTCCTGGCTGAGGGCG TCTG	AGGAGCTGGTCTCGGATCTCCCAGGCA AACAT	c.329G>A (p.R110Q)
NKX2-1-c706-pCMV3	CAGGTCGAGATCTGGTTCCAGAACCACC GCTA	AACCAGATCTCGACCTGCGTGGGCGTC AGGTGGAT	c.706A>G (p.K236E)
NKX2-1-GFP-pcDNA3.1	TAACGGCCGCCAGTGTGCTGGAATTCAT GTGGTCCGGAGGC	TTGCTCACCATGGTGGGCCAGGTCCGA CCGTATA	*NKX2-1* cDNA sequence

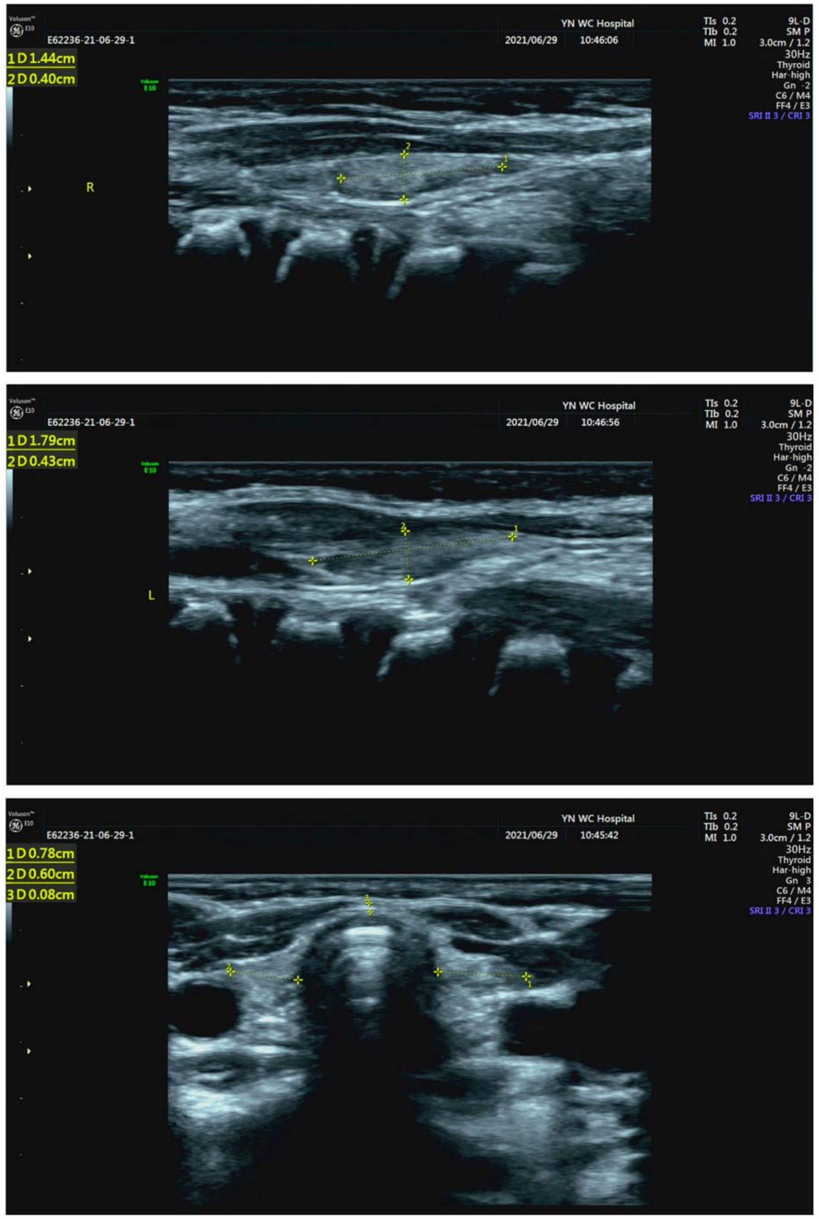

Figure S1. The thyroid ultrasound of P3. The sectional size of bilateral thyroid lobes: 1.44 * 0.78 * 0.40cm on the right (reference values for 4 years old, 2.79*0.86*0.69 cm), 0.08cm thick at the isthmus (reference value 0.14 cm), 1.79 * 0.60 * 0.43 cm on the left (reference values 2.66*0.94*0.60 cm).

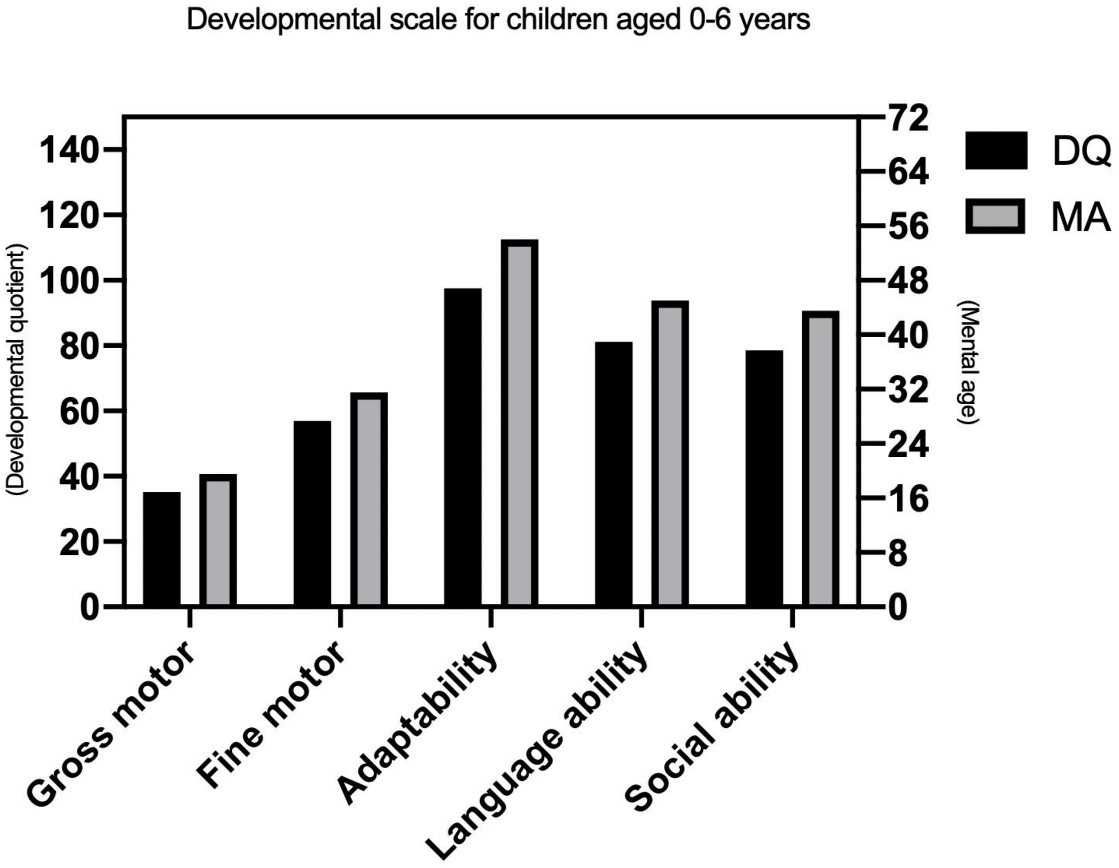

Figure S2. The developmental evaluation of P3 at 55 months. The average developmental quotient is 69.9 (The reference values of score for 0-3-year-old children, 90-115), and the average intellectual age is 38.7 months.
